# Challenging Hierarchies Through Animality: Interspecies and Gender Relations in Disney’s *Beauty and the Beast* and *The Princess and the Frog*

**DOI:** 10.3390/ani16071055

**Published:** 2026-03-30

**Authors:** Célia Jacquet

**Affiliations:** Faculty of Languages, Humanities and Social Sciences, University of Angers, ED ALL, 3l.am, 49000 Angers, France; celia.jacquet07@gmail.com; Tel.: +33-786951184

**Keywords:** Disney animation, toxic masculinity, anthropomorphism, critical animal studies, ecofeminism, gender studies

## Abstract

Through the combined lenses of ecofeminism, masculinity studies, and critical animal studies, this article examines the cultural functions of animal metamorphosis in two Walt Disney animated feature films, *Beauty and the Beast* and *The Princess and the Frog*. It argues that animality operates as a narrative and symbolic space in which dominant gender norms and human–animal hierarchies are temporarily destabilized and reconfigured. Drawing on film analysis, this study shows how the animal figure enables the emergence of alternative masculinities—sensitive, relational, and ecologically attuned—while simultaneously exposing the structural limits of this apparent subversion. Although these films challenge toxic masculinity and propose more egalitarian interspecific relationships, their narrative resolutions ultimately reinstate anthropocentric and heteronormative frameworks by reasserting human centrality and normative romantic closure. By situating Disney’s representations within broader Western dualistic logics of domination (culture/nature, masculine/feminine, human/animal), I demonstrate that animality functions less as an autonomous mode of existence than as a transitional narrative device facilitating human self-transformation. In doing so, this article contributes to current discussions on how culturally mediated representations of animals shape human social imaginaries, ethical frameworks, and understandings of interspecies relationships.

## Simple Summary

This study shows how animal characters and animal metamorphosis in Disney feature films support the rise of a new masculinity, while questioning traditional gender roles. The aim is to highlight the fact that male characters who take on an animal form become more caring, vulnerable, and equal in their relationships with the heroine on their side. At the same time, animal companions help reframe nature as something worthy of respect rather than control. It is confirmed that in doing so, the Walt Disney Company deploys transformative narratives to challenge the sharp boundary between humans and nature drawn by Western societies that treat animals as fundamentally separate from—and inferior to—human beings. While demonstrating that the presence of animals interrogates this divide that ecofeminist scholars have long criticized, this article also shows the limits of the movies’ ambivalent portrayal of non-human animals. Despite their common criticisms of humans’ violent behaviors towards animals and the natural world, they ultimately erase the animalization of the princes to restore conventional social order, suggesting that animality serves only as a temporary detour to humanity.

## 1. Introduction

After several years spent living among the Achuar, French anthropologist and ethnologist Philippe Descola asserted that nature does not exist in the sense understood by the Western philosophical tradition [1]. According to the Achuar, the very idea of nature constitutes a misinterpretation, since for them non-humans are not perceived as separate elements from humans, but, on the contrary, as “social partners” [2] with whom it is possible to communicate, notably through dreams and magical incantations. What we are used to considering as “nature”—as opposed to “culture”—would, therefore, be nothing natural, says the anthropologist who, inspired by the Achuar’s treatment of plants and animals, considers that other perceptions and relationships are possible.

The Australian philosopher and ecofeminist Val Plumwood has also been interested in the Western culture/nature polarity in her work of philosophical deconstruction. According to this pioneer of “critical ecological feminism,” a detailed analysis of dualism “shows that its characteristic logical structure of otherness and negation corresponds closely to classical propositional logic, the leading logical theory of modernity.” In her essay *Feminism and the Mastery of Nature*, she hence looked at what she calls “The Master Model”, that is the dualistic structure that organizes the world through hierarchical oppositional pairs (culture/nature, reason/emotion, masculine/feminine, human/animal), within which “the master identity is […] constructed by the systematic denial to the other group of qualities regarded as desirable or valuable in human terms.” In other words, she explores how “contrasting concepts (for example, masculine and feminine gender identities) are formed by domination and subordination and constructed as oppositional and exclusive,” allowing the repudiation of non-humans and, more generally, what Western philosophy has been calling “nature” [3]. According to her, this “hyperseparation” of nature outside the sphere of increasing mechanistic culture constitutes the very heart of contemporary ecocidal logics. For her part, radical feminist and environmental activist Françoise d’Eaubonne noted, as early as the 1970s, that “any separation reinforces a difference that it underlines and creates even if it does not exist” [4]. In other words, it is the severing of the links between humans and the Earth that is responsible for the environmental crisis that threatens life on our planet today.

This is why ecological feminism, or ecofeminism, emphasizes the importance of post-dualist and non-anthropocentric alternatives. To bridge the cultural distance that’s been symbolically separating culture from nature in our societies, it would be necessary to renew the representations of non-human animals. As American writer Charlene Spretnak would argue, it appears crucial, in our time of growing concern about non-human beings and environmental causes, to address classical dichotomous patterns and logics, while recognizing that we are “embodied and embedded in a dynamic sphere of physical relationships and processes that create real commonalities.” For it is indeed our “[f]ailure to perceive real connectedness, the curse of modernity, [that] has led us to inter- and intra-species disasters” [5]. And to quote marine biologist Rachel Carson, “[t]he history of life on earth has been a history of interaction between living things and their surroundings” [6]. It is in the wake of these critiques of the human/nature dualism that the essayist and journalist Pascale d’Erm underlines the metaphor of “reweaving the living” [7] in the creation of tomorrow’s world.

Reweaving the relationships established between humans and non-human animals is precisely what the Walt Disney Company has sought to do, in its own way, with its filmic texts. Before continuing, let us keep in mind the etymological link between “text” and “weaving” (texere); the “text” (textus) would therefore be a mode of creation perceptible as a “fabric” or a “weft.”

Since its origins, the Walt Disney Company has indeed placed the animal at the heart of its cultural and narrative identity. From the famous mouse that adorns its iconic logo to the countless creatures that populate Disney’s multimedia corporation, the animal is much more than a mere decorative motif. Often resorting to anthropomorphism—or, systematically projecting human traits onto non-human animals—this omnipresence on screen reveals the animation studio’s fascination with the porous boundaries between the human and the non-human.

If the animated classic *Bambi* [8], which perhaps best reflects Walt Disney’s concern for the destruction of nature by humans, contributed to early ecological awareness, it is in the adaptations of European fairy tales that the presence of animals proves to be the most subversive—particularly by highlighting the mechanisms of domination that govern our societies.

Drawing on film analysis and the theoretical contributions of gender studies, ecofeminism, as well as critical animal studies, this article aims to demonstrate the extent to which relationships with animals promote the emergence of new masculinities in two Walt Disney animated feature films, *Beauty and the Beast* [9] directed by Gary Trousdale and Kirk Wise in 1991, and *The Princess and the Frog* [10] directed by Ron Clements and John Musker in 2009. As will be shown, the symbolic passage through the animal world produces a temporary disarticulation of both gender and interspecies hierarchies in these fictional narratives.

I chose to study two musicals together precisely because, despite being released nearly two decades apart, they share a distinctive approach to animality: far from mere background presences, the animal characters are deeply bound to the prince figure, driving the story forward and carrying its deeper meanings. Moreover, both of these stories appear, from their very titles, which place humans and animals side by side in a programmatic conjunction, to echo Donna Haraway’s argument that “species of all kinds, living and not, are consequent on a subject- and object-shaping dance of encounters” in which “all the dancers are redone through the patterns they enact” [11]. As we shall see, this intermingling of species in the fantasy narrative is likely, by projecting other possibilities, to “circumvent or subvert the norms in force,” to cite fantasy and literature scholar Anne Besson [12].

The article, therefore, examines how engagement with animals contributes both to the deconstruction of Plumwood’s “Master Model” and what d’Eaubonne has famously called the “Male System”—the patriarchal domination and the exploitation of both nature and women—and to the establishment of new relational and narrative paradigms between humans and non-humans in these two mainstream animated films closely aligned with “eco-fiction” [13]. Traditionally relegated to the realm of radical otherness in Western thought, I shall argue that the non-human animal becomes a potential catalyst for social transformation by paving the way for more egalitarian relationships between both sexes and species.

## 2. From Beast to New Man: Animal Metamorphosis as a Symbol of an Alternative Masculinity in the Making

Screenwriter Linda Woolverton modernized Madame Leprince de Beaumont’s tale *La Belle et la Bête* [14] in *Beauty and the Beast*, telling the story of Belle: a young French woman who feels misunderstood and cramped in her small village during the Ancien Régime. Rather solitary and passionate about reading books, the main protagonist finds it difficult to mingle with others and spends her days dreaming of adventures, while tirelessly rejecting the advances of Gaston, the local seducer with a macho character.

Her monotonous life takes a new turn when her father becomes the prisoner of a monster residing in a huge castle deep in the woods. However, as the prologue reveals, this monster is in fact a cursed prince. After refusing hospitality to a fairy disguised as a wretched old woman, the young prince, selfish, angry, and arrogant by nature, has been transformed into a hideous beast. But in addition to this metamorphosis that is supposed to reflect the ugliness of his heart, the prince is also condemned to die on his twenty-first birthday, unless he manages to make himself truly loved, despite his repulsive appearance. Unaware of the enchantment, Belle offers to switch places with her ill father. During her stay at the castle, not only does she marvel at the various living objects and furniture that populate the gothic setting, but Belle also learns to see beyond appearances. Several arguments and reconciliations later, she ultimately falls in love with the master of the house and manages to lift the curse that weighed on him, his castle, and his staff.

Admittedly, the staging of a relationship “between a gentle, devoted, understanding woman, and a tortured and/or violent man whom she seeks to save” [15] is not without suggesting a certain trivialization of sexual domination [16,17], even a romanticization of the Stockholm syndrome. Yet, as we shall see, the invented narrative tension between Belle’s suitors in Disney’s adaptation of Leprince de Beaumont’s 1756 tale draws on the rising influence of Men’s Studies to criticize traditional models of masculinity. In the wake of feminist criticism, this field of research, also known as Masculinity Studies, has been focusing on shedding light on the constructed nature of masculinity. With a sociocultural origin, masculinity cannot be reduced to the canonical heteronormative representation of the “real man,” as if there were an original identity of the man or a “true masculinity [...] thought to proceed from men’s bodies—to be inherent in a male body or to express something about a male body” [18].

According to American philosopher Judith Butler, gender indeed “ought not to be construed as a stable identity or locus of agency from which various acts follow; rather, gender is an identity tenuously constituted in time, instituted in an exterior space through a stylized repetition of acts” [19]. Following the Butlerian argument, French philosopher Olivia Gazalé offers a critical analysis of virility, which she describes in her essay *Le Mythe de la virilité* as “an imaginary cultural construct” deeply rooted in “male cosmogony” [20]. A male cosmogony that has been in part shaped by violence and destruction. Inspired by the work of sociologists Pierre Bourdieu [21] and Connell, the expression “toxic masculinity” [22,23] refers to this specific model of virility, oriented towards domination and control. As sociologist Michael Flood notes, “the adjective ‘toxic’ emphasizes the fact that it is only a particular form of masculinity that is targeted, thus governed by a set of unhealthy, harmful, restrictive, dangerous norms and practices, etc.” [24]. Popularized across the Atlantic in the 2010s, the term is used to make sense, among other things, the motivations behind certain mass killings and the “rape culture” [25] that is rampant on campuses and in mainstream cinema.

In *Beauty and the Beast*, this model of virility is embodied by the film’s main antagonist, Gaston. Absent from the original eighteenth-century tale, this macho seducer with the voice of a tenor was created from scratch by the Walt Disney Company to serve as a rival to the Beast, against whom he competes for the favor of the female protagonist, whom he insists on marrying despite her repeated refusals.

Did screenwriter Linda Woolverton invent this “masculinist” [26,27] character to make young audiences more aware of the dangerousness of certain behaviors? The question arises, especially in the face of the staging of Gaston’s marriage proposal to Belle. Filmed entirely from the heroine’s point of view, this scene orchestrates less a romantic act than a thinly veiled metaphor for sexual harassment. Indeed, from the moment the male character enters the screen, everything is done to emphasize his coercive attitude: he imposes himself on Belle’s very intimacy by forcefully entering her home. The low-angle shot used to film Gaston’s approach not only accentuates the predatory dynamic between the two characters and emphasizes his domineering nature, but also visually eliminates Belle’s escape routes: Gaston deliberately moves a chair to block the door to an adjacent room, while the front door itself disappears, relegated off-screen. A backward tracking shot then follows Belle’s retreat as she recoils, ultimately trapped against the front door in the arms of the hunter, who remains determined to ignore both her lack of consent and her visible discomfort.

In doing so, the film resonates with feminist denunciations in the 1970s about the general glamorization of gender violence. Molly Haskell states in her book *From Reverence to Rape: The Treatment of Women in the Movies* that “violence is the indispensable staple of male pornography, expressing itself in apocalyptic allegories of male virility” [28]. For her part, ecofeminist thinker Susan Griffin questions what she considers to be an American specificity. She links together the glorification of war by American culture, the attraction of men to violence, and the very act of rape, which she describes as “a perfect combination of sex and violence” [29]. Since heterosexual eroticism feeds on male domination and female submission, she then concludes, “not only does our culture teach men the rudiments of rape, but society, or more specifically other men, encourages the practice of it” [30]. *Beauty and the Beast*, however, works to condemn this rape culture by discarding the conventional androcentric perspective in favor of a feminine, even feminist, one. To highlight the oppressive situation the heroine experiences, the film relies on body language, focusing on Belle’s face as Gaston attempts to force a kiss upon her. Not only does this focus intensify her sense of entrapment, but it also frames the non-consensual kiss as attempted sexual assault. Another close-up of Belle’s hand desperately searching for the door handle may even signal the production company’s deliberate effort to break from the classical fairy tale formula, in which the prince effortlessly enters the princess’s bedroom (whether literal or symbolic) and carries her off to his castle to live “happily ever after.” In this manner of favoring the victimized heroine’s experience, her visible disgust and clear lack of consent, this cinematic lesson on consent seems to implicitly align with feminist concerns.

Moreover, what particularly stands out in this family-friendly film is how eloquently the animal motif reflects shifting social attitudes toward specific gender behaviors. Through comedy, the animal is used as a cipher to criticize the logic of male domination that Gaston embodies. This association operates primarily through visual and burlesque means. Set to a triumphant fanfare, Gaston finds himself stuck in a mud puddle, ridiculed by a pig perched on top of his head that crushes him with its full weight. The scene’s humor derives from a reversal of interspecies power dynamics: neutralized by an animal traditionally associated with filth and clumsiness, the self-proclaimed paragon of hegemonic masculinity is reduced to a posture of humiliation and powerlessness.

Even more significantly, this symbolic association with the animal extends beyond mere comic relief to critique toxic behavior. The pig functions as an implicit metaphor for Gaston himself. In the Western cultural imagination, the term pig connotes rudeness, boorishness, and moral reprehensibility. Such attributes resonate with the hunter’s predatory pursuit of Belle. By such means, the animal association enables a broader critique of the masculinity embodied by the hunter. This reading acquires resonance in the contemporary French context, where the “MeToo movement”—launched in 2006 by Tarana Burke to encourage survivors to talk about their sexual assault [31] was translated as “Balance ton porc” (literally, “denounce your pig”). While it is not my purpose to anachronistically impose a contemporary activist framework on Disney’s 1991 film, I want to shed light on how this semantic convergence reinforces Gaston’s embodiment of predatory masculinity, whose public humiliation serves as an implicit condemnation of gendered domination.

Following Carol Adam and Lori Gruen’s statement that ecofeminism “addresses the various ways that sexism, heteronormativity, racism, colonialism, and ableism are formed by and support speciesism” [32], I argue that this fictional politicization of the animal character highlights a cross-relationship of domination. Indeed, as the words of Gaston’s sidekick in the movie suggest, the animal makes it possible to bridge the gap between different forms of domination that are both patriarchal and speciesist: “Wow, you didn’t miss a shot, Gaston. You’re the greatest hunter in the whole world! No beast alive stands a chance against you. And no girl for that matter.” The domination of women and the domination of animals proceed from the same imaginary of possession. “I’ve got my sight set on that one,” says Gaston when Belle appears in his field of vision. Adopting the hunter’s point of view briefly, the camera then hastens to show us, via a quick tracking shot followed by a shot-reverse shot, what he points to with the tip of the rifle that was used to shoot down a moorhen in mid-flight a few moments earlier. Obsessed with the conquest of women as well as animals, the hunter embodies the destructive ideal of classic masculinity, based on domination, control, and the eradication of all otherness [33]. Such virility is not limited to the sexual intimidation of Belle, who is considered a mere “wife/trophy” [34], but extends to speciesism with the programmed killing of the Beast. In another scene, the presence of animals on screen once again brings up his status as an oppressor. Positioned atop a pedestal composed of both animal flesh (carcasses hung on the walls and skin serving as a carpet) and feminized bodies (bubble-brained young women reduced to mute admiration), Gaston is presented in this feminist film as the nexus of an ideology of total possession of the “Other.” Far from the hero he claims to be, this invented character even becomes the story’s true monster. Historian Marina Warner aptly observes that Gaston takes the part of the “real” beast, embodying social deviance, ecological predation, and patriarchal domination. During the final deadly fight between the Beast and the hunter, close-up shots and lightning flashes dramatically emphasize the latter’s true villainous identity. Rooted in a cinematic tradition of Gothic horror where facial disfigurement signals moral corruption, his distorted face becomes the site of monstrous transformation, contradicting his initial image as a virile seducer.

Beyond its critique of toxic masculinity, the animal also functions as a subversive locus for alternative paradigms of gender representation. Grounded in the socio-cultural context of the 1990s—a period deeply shaped by queer theory and by a growing reassessment of the gender models circulated on screen—the film complexifies the archetypal prince through the hybrid figure of the Beast to push forward a renewed form of masculinity. One that would be more attuned to contemporary ecological and feminist concerns. While the hunter reveals himself to be an abject individual, the Beast—though initially angry and violent—evolves toward redemption. If his physical appearance is, at first, repulsive, the story reveals the kindness hidden behind the monstrous mask. For the Beast, despite his repulsive appearance, is capable of love, clemency, and sacrifice. While Gaston dreams only of locking Belle into a subordinate domestic role, even using manipulation and blackmail to achieve his ends, the Beast, on the other hand, is ultimately much less oppressive: ready to sacrifice all his chances of regaining a human appearance, he even lets his beloved go. Unlike Gaston, he softens over the course of the story and ultimately learns, alongside Belle, to become a new kind of man. Less driven by anger, the Beast discards his aggressive behavior to reconnect with his environment, his household staff, and the fragile birds he attempts clumsily to feed with his large, clawed hands. This pivotal shift is visually rendered through a shot-reverse shot filmed from Belle’s perspective, set against cheerful music whose lyrics celebrate the prince’s surprising gentleness and even innocence. Contrary to Gaston’s aggressive methods, the Beast comically fears injuring the small birds so much that he remains still in their presence, transforming himself into a harmless perch for them. Under the guidance of Belle and the other anthropomorphized characters—whose presence provides both entertainment and emotional solace—the Beast embraces an ethic of care [35], breaking free from the hegemonic model of masculinity embodied by the destructive hunter.

For cultural studies scholar Simon Massei, this masculine dichotomy reflects a crucial shift in the social and cultural perception of “good” masculinity: “Excessive musculature, greed, stupidity, misogyny: the model of the stupid, boorish and brawling man emerges as the primary counter-model of masculinity” [36]. Departing from Gaston’s “excessive virility,” the beastly prince is charming the heroine because of his gentleness. To better emphasize this gap between the two male characters, the film creates a completely different natural setting. While the storm and lightning exacerbate Gaston’s violence in his final appearance on screen, the more domestic setting of the castle garden, with its joyful music and the chirping of the colorful sparrows, tends to visually reveal the softness of the Beast, now closer to the “sensitive man” [37] than to the classic conquering warrior. As Warner states, “[...] self-styled heart throbs who fancy themselves Supermen are now the renegades, and wild men in touch with nature and the beast within the exemplars.” Disney’s animals thus confront traditional representations of violent and warlike masculinity—typical of male heroes in action films of the 1970s and 1980s, with the very muscular actors Arnold Schwarzenegger and Sylvester Stallone in the lead. Instead of piercing the birds’ bodies with a long rifle or forcing Belle into submission, the Beast crouched down by her side to play with the other animals in the forest. The crushing low-angle shots give way to wide horizontal shots, as if to better resonate with Greta Gaard’s reminder of the compatibility between masculinity and ecology [38]. While he initially appears capricious and arrogant, the animalized prince opposes Gaston’s virility by seeking a genuine connection with Belle, whose influence encourages sensitivity, romanticism, and self-sacrifice. To become a better man, in this film, is also to become more attentive and caring toward those who are more fragile. In doing so, Disney’s treatment of masculinity proves inseparable from a broader ethical repositioning, as such a composite character, simultaneously human and non-human, foregrounds a new model of masculinity grounded in interdependence. The departure from traditional heroic models is epitomized in the resolution of the curse: for the first time in a Disney princess film, it is the prince who is saved, freeing the Beast from his fatal enchantment.

Through its various animal representations, contrasting an oppressive patriarchal figure with a decidedly more “charming” fantastic Beast, the 1991 film demonstrates how the Walt Disney Company remained attentive to evolving social mores. Like the feminist writers and poets of the 1960s and 1970s—a period of social upheaval marked by the emergence of second-wave feminism, inseparable from the counterculture movement and the imperative to revisit Western culture’s foundational narratives—the studios applied in their own way new critical lenses to existing tales by interrogating and deconstructing entrenched gender stereotypes.

This subversive approach would continue nearly two decades later in *The Princess and the Frog*, where interspecies encounters catalyze a transformation of social paradigms.

## 3. Back to the Wilderness: Animality to Say Equality

After Madame Leprince de Beaumont, the Walt Disney Company once again drew from the fairy tale tradition and its characteristic menagerie of animals and non-human beings. The animation studios decided to adapt the Brothers Grimm’s *Der Froschkönig oder der eiserne Heinrich*, first published in 1812 [39], to weave a story in which animality prompts a significant reconfiguration of social relations. Magically transformed into frogs by an evil sorcerer, the two human protagonists—Tiana, a Black waitress from New Orleans, and Prince Naveen from the imaginary kingdom of Maldonia—are forced into exile in the heart of the bayou, where the voodoo priestess Mama Odie resides. More than a luminous character whose close relationship with the bayou wildlife contrasts with the antagonist’s destructive behavior, she is also reputed to be the only one capable of helping the protagonists lift the metamorphosis curse. Alongside typically anthropomorphized animal characters—such as Louis, a cheerful trumpet-playing alligator who comically thinks he’s Louis Armstrong, and Ray, a very romantic glowworm of Cajun origin—Tiana and Naveen reluctantly embark on a perilous journey into the heart of Louisiana’s iconic swamps to regain their human form.

Though structured as a romance between the two protagonists, the film ultimately examines human/non-human encounters through the lens of equality. Indeed, humans, nature, and animals coexist in this resolute “greener” cartoon that broke away from the original tale as both Tiana and Naveen transform into frogs whose survival depends on their capacity to forge bonds with the bayou’s non-human community. In turn, Louis the alligator and Ray the glowworm become crucial companions in the humans’ quest for liberation.

Through this plot twist, *The Princess and the Frog* challenges what ecofeminist philosopher Karen J. Warren has called “sexist-naturist language,” that is, “a language that depicts women, animals, and non-human nature as inferior to (having less status, value, or prestige than) man and male-oriented culture” [40]. Here, the film effectively distances itself from the “feminine ideal” myth, no longer placing the feminine on the side of the “inferior,” the “secondary,” or the “inhuman,” by creating a fantasy universe where the animalization of the female body goes hand in hand with that of the male body.

Not only does this shared metamorphosis disrupt sexist animal metaphors and expressions that describe women pejoratively and in sexualized terms [41], but it also enables the emergence of a new social order in which the two future lovers are positioned primarily as equal partners. Following their comedic first encounter—far removed from the saccharine narrative conventions the studio had long established—the two protagonists strike a bargain: Tiana will serve as a matchmaker between the prince and her friend Charlotte, a wealthy Southern belle purportedly capable of breaking the curse with her kiss; in exchange, Naveen will provide financial backing for her restaurant venture. Subverting narrative conventions, the protagonists start their journey as business partners before turning into romantic partners. This fundamentally strategic and transactional relationship breaks from the traditional love-at-first-sight trope, often used in Disney princess films. And to further avoid narrative immediacy, the prince’s screen time is expanded, permitting romantic feelings to develop gradually through their common journey toward freedom. Unlike the master narrative of the charismatic hero capable of defeating all kinds of monsters and perilous situations on his own, *The Princess and the Frog*’s innovative storyline insists on the need for the characters to stand together as they are confronted with the same ordeals, the curse, which serves as a disruptive element, and the adventures that follow.

To stage this new partnership, the artistic teams rely on traditional cinematographic representations of the famous bayou. As film researcher Taïna Tuhkunen explains, “[i]n the southern wilderness, nature rarely allows humans to recharge their batteries, and the myth of beneficent nature is regularly and spectacularly undermined” [42]. The infamous swamps do indeed appear to be a wild, dangerous, and terrifying territory, almost a “paradise for reptiles and other antediluvian or subtropical species [where] the white man struggles to control plants and beasts.” Plunged into darkness and infested with mosquitoes, they evoke gloom, filth, and stagnation, which no doubt partly explains its success in “the genre of horror where the Cajun culture remains profoundly anxiety-provoking,” Tuhkunen writes. The Disney animated film’s very first images of the iconic bayou engage with this imaginary by presenting a sinister and threatening wilderness as the two little frogs almost get devoured and crushed by the various creatures that inhabit the disturbing and dreadful dark marshes, portrayed in every shot as an “uncontrolled and even uncontrollable” space, “the place of wild beasts” [43]. To emphasize this high risk of death, the pestilential territory gains in dramatic intensity as night falls. The atmosphere indeed becomes ghostly with the eerie soundtrack that keeps the viewer on the edge of their seats.

Yet, behind its traditional allure, this stereotypical cinematographic representation of both an appalling and fascinating New World is much more subversive than one might think, since it upsets the traditional division of gender roles. Unlike European settings, embodied on screen by castles and rural villages, where social and gender codes remain operative, the American wilderness does not recognize the status of the prince or the conventions of femininity. Stripped of his social privileges in this unpredictable environment, Prince Naveen, accustomed to the comfort of court life, must now learn domestic skills, such as cooking, traditionally associated with the Disney heroines of the golden era, alongside Tiana.

Rather than conforming to the conventional fairy tale pattern in which the prince remains an underdeveloped secondary figure who appears at the story’s conclusion to deliver a happy ending [44], *The Princess and the Frog* uses Naveen’s animal form to propose an alternative model of gender dynamics. Indeed, his amphibious transformation strips away the heroic masculine attributes typically associated with Disney princes, such as the physical strength and protective capacity exemplified by Prince Philip vanquishing the dragon in *Sleeping Beauty* [45] and Prince Eric battling Ursula in *The Little Mermaid* [46]. Instead, when confronted with the bayou’s dangers—alligators, hunters, and encroaching darkness—Naveen doesn’t possess his predecessors’ super strength but instead exhibits the same helplessness as Tiana. Both protagonists scream, flee, and seek shelter when faced with predators. Their diminished bodies render physical confrontation with the alligators and the human hunters impossible, for example, leaving them equally defenseless and imperiled. This shared powerlessness contributes to the dismantling of traditional gendered division, where brute force is coded as masculine and vulnerability as feminine, replacing the conventional dynamic of male protector and female protected with a model of mutual dependence and egalitarian cooperation in navigating the heroic journey. Beyond its dramatic effects, the animal presence onscreen hence precipitates an egalitarian redistribution of gender-specific characteristics that become shared attributes.

Indeed, *The Princess and the Frog* systematically blurs the norms by turning the prince into the masculine side of the archetype of the “damsel in distress.” When Naveen, perched on top of a tree trunk to escape hungry alligators, begs Tiana to throw a creeper to rescue him, the scene ironically summons the Rapunzel figure (which the Walt Disney Company would adapt for the screen a year later under the title *Tangled* [47]). Similarly, in a later scene, Tiana wakes Naveen up by throwing an acorn over his head before mockingly calling him “Sleeping Beauty.” Through these parody effects, the film does not content itself with narrative innovations but also becomes a sort of palimpsest in which conventions become a comic and self-reflexive springboard. In doing so, the Walt Disney Company thus demonstrates, through animal characters, its ability to challenge established norms, to question its own codes with humor, and to reinvent the archetypes of its enchanted universe.

By the same token, animalization prefigures another transformation in the relationships between species. While serving a typical role of comic entertainment, the alligator Louis and the glowworm Ray accomplish a much more ambitious narrative function: to decenter the anthropocentric gaze and propose a more ecologically sensitive apprehension of interspecific relationships. Indeed, far from being mere foils for metamorphosed human protagonists, these animal companions become essential guides to rediscover the Louisiana environment. Ray, with his joyful “going down the bayou” song and his intimate knowledge of the swamps, embodies an Indigenous voice that reorients the narrative. It is no longer the human gaze that dominates and names the natural space, but an animal perspective that reveals the intrinsic richness and beauty of an endangered ecosystem.

In other words, the film employs a strategic shift in visual perspective that challenges anthropocentrism while inviting viewers to reconsider humanity’s bond to the natural world by showing it in a different, animalized light. When Tiana and Naveen enter the bayou as frogs, the film’s cinematography adopts their diminutive scale, fundamentally altering the audience’s perception of both the animal protagonists and their environment. This technique moves beyond human-centered perception by inviting viewers to adopt a non-human perspective. As in Anthony Wilson’s essay *Shadow and Shelter: The Swamp in Southern Culture* [48], the Walt Disney Company offers two different approaches to the bayou. On the one hand, the film paints a rather conventional portrait of a natural environment that is both hostile and distressing. But this representation, very much in the manner of North American horror films, is superimposed on a completely different image of the Louisiana swamps, which the film reminds us of being prey to destruction by the incorporation of ridiculous, but no less dangerous, hunters who seek (literally) to make the skin of frogs and alligators. By centering on the links between humans and non-humans, this filmic adaptation of an old tale proves to be more environmentally committed. It notably raises awareness of the precarious situation of the Louisiana marshes. Rather than merely a disturbing space associated with otherness, the bayou is now represented as a natural environment teeming with life and wonders in need of protection.

To highlight its beauty, the film literally magicalizes the disturbing bayou through shimmering colors and the rhythm of jazz music—another marker of the American South—ultimately transforming it into a magnificent spectacle. Sublimated by a wide shot, the brackish waters become a kind of inverted mirror: through their reflections, they appear capable of magnifying the magic of the marshes tenfold. Reminiscent of fairy dust, the glowworms provide an enchanting illumination that envelops Tiana and Naveen in an aura of magic. Through this lighting effect that seems to mirror the very multiplicity of nature, the film endows Louisiana’s swamps with a dreamlike quality, transforming a seemingly hostile space into a site of infinite wonders conducive both to escapism and transformation. As Jonathan Burt notes, “animal imagery does not merely reflect human–animal relations and the position of animals in human culture but is also used to change them” [49].

By divesting the bayou of its inherently fetid and unhealthy imagery, the film turns the wilderness into a marvelous space where new bonds between humans and nature are possible. Despite the expected revival of the enduring ideal of marital happiness, the final sequence is endowed with a distinctly “greener” hue than in the tale on which it is based. The expected wedding ceremony now takes place in the heart of a singularly luminous bayou. While film scholar Eve Benhamou identifies a form of aesthetic and generic nostalgia capable of both revitalizing traditional Disney romance and subverting its gender and racial constructions [50], I further contend that this reinvention also extends to interspecies relationships. Admittedly, the film once again concludes with yet another heteronormative marriage. Nevertheless, it remains an optimistic eco-film in the way it represents, through its verdant promises, the union between the human world and the realm of wild nature, in a wide shot that frames the couple surrounded by the inhabitants of the bayou.

In doing so, this happy ending celebrates new forms of coexistence that do not stop at marriage, unlike previous Disney adaptations of fairy tales. It instead extends to the presence of cheerful animals who are welcomed into the restaurant that Tiana becomes the owner of, and whose very name, Tiana’s Palace, reads as an ironic confirmation of the film’s refusal to submit to the codes of yesteryear. Let’s also note how Louis, who brings to the screen a species threatened with extinction in the 1920s and 1930s, ultimately fulfills his dream by performing on stage alongside a human jazz band.

By taking considerable liberties with the old frog prince of the Brothers Grimm fairy tale, the Walt Disney Company stages the possibility, if not the necessity, of reintegrating nature into culture, thereby continuing, in its own way, the dismantling of the Western culture/nature dualism through this ecofeminist musical.

While carrying a subversive potential, this interspecies fusion confronts the problem of instrumentalization. Indeed, by relying on personification and preserving established character typologies and conventional narrative structures, it may, on the contrary, turn the animal into a conduit for our normative systems.

## 4. The Ambiguous Place of the Animal Character: Between Ruptures and Normative Persistence

At the end of our analysis of *Beauty and the Beast* and *The Princess and the Frog*, it appears that the animal occupies an ambivalent position in Disney’s reworking of traditional narratives. On the one hand, animal metamorphosis condemns toxic masculinity through the rejection of the aggressive virility model embodied by Gaston and the valorization of a sensitive masculinity in the Beast. On the other hand, the passage through the Louisiana wilderness encourages a redistribution of gender roles and a partial recognition of the agency of non-human characters. Nonetheless, these narrative updates encounter structural limits that call for further examination.

John Berger’s reflections on the specificity of the human gaze shed light on the unexamined foundations of Disney’s anthropomorphism. According to Berger, “by no other species except man will the animal’s look be recognized as familiar. Other animals are held by the look. Man becomes aware of himself returning the look” [51]. Paradoxically, it is this very awareness of a gaze that knows it is being returned and recognizes itself in the other—that limits the subversiveness of the two films’ bonding between human and non-human characters. Although Disney’s animals, as Paul Wells notes, interrogate both humanity and animality [52], they also serve as narrative cyphers for broader social concerns while illustrating, through fiction, our social systems without fundamentally decentering the human gaze that structures their representations. Indeed, if animated characters are “phenomena” capable of occupying multiple representational positions at once, beast and human, subversive and innocent, then Disney’s animal figures do indeed carry radical potential. Animal characters function as catalysts for the transformation of gender and species hierarchies as they destabilize conventional gender roles and encourage egalitarian interspecies relationships. Yet these reconfigurations remain dependent on the anthropocentric gaze Berger describes: the animal continues to exist primarily as a reflective surface through which humans “become aware of themselves.”

As conventional endings of both films ultimately foreclose it, while the passage through animality enables a questioning of male domination, it also paradoxically reinscribes the characters within a heteronormative narrative framework. In *The Princess and the Frog*, amphibious form alone proves insufficient to depart from Disney’s filmic conventions. This is particularly evident when Tiana and Naveen, despite their animal appearances, engage in a traditional romantic waltz in the bayou, now portrayed as an enchanted setting reminiscent of older Disney princess films. Like the crab Sebastian in *The Little Mermaid*, Ray the glowworm orchestrates the sequence as a theatrical master of ceremonies: as he serenades Evangeline, the star he’s in love with, the two frogs twirl beneath the dim lights of the bayou, reproducing a “naturalized” version of aristocratic ballroom choreography. Far from breaking with the visual and choreographic codes of early Disney films, this sequence transposes the castle into the wilderness, suggesting that even when metamorphosed into animals, the protagonists remain bound by courtly rituals and the narrative expectations of heteronormative romanticism.

Furthermore, the conventional marital ending is rendered possible by the ultimate erasure of animality itself. In the final denouement of *Beauty and the Beast*, the spell lifted by Belle’s tears restores the prince’s human form, literally eclipsing the non-human animal and the bestiality that had nevertheless enabled the relational transformations between Belle and the Beast. Even when non-human characters remain in *The Princess and the Frog*, their presence is ambiguous, raising the question of what truly remains “animal” in these personified representations. This is particularly evident in the character of Louis, the jazz-loving alligator who performs on stage in ways indistinguishable from his human counterparts. Presented as the ultimate horizon of happiness, the return to a human and heteronormative order thus seems to support the idea that animality functions only as a temporary detour; a transitional phase leading to the reaffirmation of the very social and species hierarchies it initially appeared to contest.

As a result, such narrative resolutions reveal Disney cinema’s difficulty in fully escaping Plumwood’s “Master Model.” Although *Beauty and the Beast* and *The Princess and the Frog* attempt to deconstruct certain forms of male domination by proposing alternative models of masculinity—sensitive, vulnerable, capable of care—they fail to fundamentally challenge anthropocentrism, or the centrality of the human within the world order. The animal seems to remain a medium, an obligatory passage toward “true” humanity, rather than a form of existence recognized as intrinsically valid.

While these films clearly participate in a feminist critique of gender relations, they struggle to extricate themselves from the dualistic framework that Plumwood identifies as foundational to Western logics of domination. Fantastic metamorphosis, far from offering true hybridity in *The Princess and the Frog* or a lasting cohabitation between human and non-human forms of life in *Beauty and the Beast*, ultimately appears as a temporary narrative device which, once its cathartic function has been fulfilled, must be erased to allow for the reestablishment of anthropocentric order.

In other words, while participating in the “animal turn” [53], this symbolic bond with the animal also raises the thorny question of the ambiguous role of the personified animal in the perpetuation of the Walt Disney Company’s normative model. Indeed, by projecting human values and behaviors onto the animal characters, the films risk reducing their otherness to an instrumental function in the service of the human narrative. In humanizing the non-human, the famous animation studios indeed tend to reiterate, under the guise of transgression, the very hierarchies they claim to deconstruct in their films, in which the animal plays a crucial narrative and symbolic role.

It would nevertheless be reductive to view these works of fiction as mere reaffirmations of normative structures. The tensions that run through them—between subversion and conformism, critique of hierarchies and reaffirmation of established orders—testify to the contradictions inherent in a mass cultural industry seeking to reconcile narrative innovation with commercial profitability, political commitment, and family entertainment. *Beauty and the Beast* and *The Princess and the Frog* thus remain significant milestones in the evolution of Disney’s representational politics in proposing alternative images of masculinity and interspecies relationships, even if these openings remain partial and ambiguous.

## 5. Discussion

At a time of growing eco-anxiety and increasing feminist awareness, revisiting these two family-friendly classics feels all the more timely: rather than compounding the despair of a world grappling with environmental crisis, they offer, however imperfectly, an optimistic vision of human-animal re-bonding while inviting men to shed the outdated models of virility that our societies are slowly learning to leave behind. Beyond their contribution to gender equality, these films also foster a more empathic relationship with the animal world. By condemning male violence, they implicitly advocate for a more compassionate treatment of non-human animals.

This is perhaps most powerfully conveyed in the harrowing final scene of *Beauty and the Beast*, during which Gaston brutally assaults the Beast. As the Beast collapses, mortally wounded, a tender, elegiac orchestral score accompanies Belle’s distress. Her gentle and careful touch of the Beast’s face as her tears blend with the raindrops falling on her stages a love that knows no species boundary, implicitly urging its audience to extend the same tenderness to the non-human lives that surround them in the real world. Echoing *Bambi*’s legacy, which contributed to a shift in perspective on nature from a dangerous adversary to a helpful partner [54], *Beauty and the* Beast deepens this emotional repositioning toward the non-human world: Belle’s devastation at the Beast’s fatal injury becomes a lesson in empathy, one that gently urges us to develop a sense of care and responsibility toward nature and animals. *The Princess and the Frog* extends this ethical gesture into Ray’s melodramatic funeral scene that leaves no doubt as to the value of non-human lives. Ruthlessly killed by the film’s villain, Ray is mourned by Louis, Tiana, and Naveen with a gravity that refuses to treat a non-human life as expendable. Rather than treating the animal’s passing as a minor narrative event, the film dedicates a whole sequence to treating the death of this side character with the solemnity of a hero’s farewell, from Louis’s mournful trumpet tribute to Naveen and Tiana’s tender gesture of laying flowers, all bathed in the ethereal glow of the glowworms. Refusing to rush past this loss, *The Princess and the Frog* takes the time to allow Ray’s companions, as well as the audience, to grieve fully, making a quiet but powerful argument that even the smallest life deserves to be mourned beautifully.

Taken together, these scenes reveal that Disney’s most subversive gesture may not simply lie in its reworking of gender roles, but also in its insistence that non-humans are urgently worth protecting and respecting.

## 6. Conclusions

It is concluded that through fantasy fiction, Disney filmic production imagines subversive ways of coexistence and reciprocity between humans and non-humans that eventually support ecofeminist forms of gender dynamics while disrupting oppressive models of masculinity. In doing so, these two musicals pave the way to a renewed relationship with animals that help reshape men’s relationship with others (women, children, and non-human animals) in our actual society, with increased care and empathy.

## Data Availability

The data created in this study are available on request from the corresponding author.
